# Thin fabric pressure sensors and TinyML smart gloves for edge IoT

**DOI:** 10.1016/j.isci.2026.116750

**Published:** 2026-08-01

**Authors:** Tuan Nghia Nguyen, Chi Cuong Vu, Viet Hoang Nguyen, Van-Ca Phan

**Affiliations:** 1 Faculty of Electrical and Electronics Engineering, Ho Chi Minh City University of Technology and Engineering, Ho Chi Minh 700000, Vietnam

**Keywords:** fabric pressure sensors, tiny machine learning, smart wearable system, edge IoT, real-time monitoring

## Abstract

Wearable human-machine interfaces require flexible sensing, low-latency processing, and secure data handling for practical edge IoT applications. This study presents a smart glove integrating thin carbon nanotube (CNT)-fabric pressure sensors with embedded tiny machine learning (TinyML) models on an ESP32S3 platform. The sensor achieves a sensitivity of 0.46 kPa^−1^, a response/recovery time of 60/45 ms, and stable operation over 3,500 cycles. Pressure signals from three fingertip channels are processed locally using lightweight random forest and k-nearest neighbors models, achieving classification accuracies of 92.1% and 96.55% with inference times of 0.185 and 4 ms, respectively. All raw sensor data remain on the embedded device, while only prediction labels are transmitted through Wi-Fi for remote monitoring. The proposed framework demonstrates a compact and privacy-aware wearable platform for real-time human-machine interaction and embedded healthcare systems.

## Highlights

• Thin CNT-fabric pressure sensors enable fingertip wearable pressure sensing
• TinyML models run on ESP32S3 with 0.185–4 ms inference
• On-device inference reduces raw data transmission in edge IoT
• Smart glove achieves up to 96.55% object classification accuracy

## Graphical abstract

**Figure:**
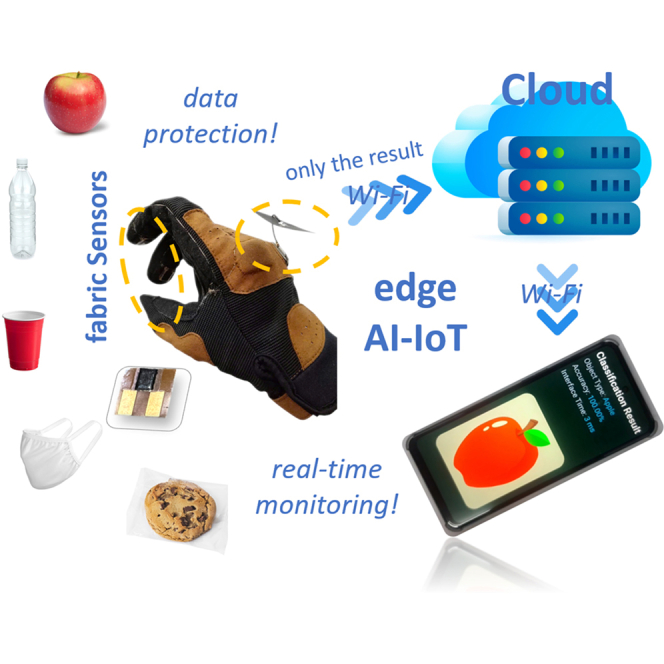

## Teaser

Applied sciences; Machine learning; Sensor system

## Introduction

The field of smart wearables^1^^,^^2^^,^^3^ is developing very rapidly, with many applications from industry to daily life. Some prominent trends and technologies include multi-touch/gesture/biometrics, voice recognition, natural language communication, virtual reality, augmented reality, extended reality (VR/AR/XR), smart cars, industry 4.0, direct brain-machine communication, medical devices, and e-healthcare.^4^^,^^5^ Wearable devices are transitioning from screens and buttons to intelligent, natural, and multi-channel interfaces—where humans and machines communicate seamlessly through voice, gestures, eyes, and even brain signals.^6^^,^^7^ Despite rapid progress, wearable systems still face challenges, including accuracy, latency, scalability, user comfort, adaptability, data security, and cost. Among these, the most significant difficulty is creating a natural experience and ensuring accuracy, safety, and reliability. There are many approaches to solving the aforementioned problems. Some studies improve AI algorithms and optimize hardware.^8^ Others prioritize natural experiences when developing innovative materials and soft sensors for simple, intuitive, and comfortable interfaces.^9^ Many researchers enhance data collection and biometric encryption to ensure user security and privacy.^10^ Achieving significant progress requires the integration of advanced technologies (such as AI, new materials, and energy) with a human-centered approach.^11^^,^^12^ However, few studies have simultaneously addressed all these aspects.

The concept of flexible sensor-edge AI refers to approaches in both directions of flexible soft sensors and embedded AI algorithms for wearables in IoT systems. In particular, some types of soft sensors have been proposed: pressure, stretch, or self-powered sensors.^13^^,^^14^^,^^15^^,^^16^ Most of these sensors are made from flexible materials, capable of stretching or withstanding deformation, such as elastic fabrics, silicon, or natural materials. These materials provide good bio-contact, easy integration, suitable sensitivity, low latency, and are suitable for body interactions such as touch/slide gestures. In addition, embedded AI algorithms include lightweight models such as random forest (RF), decision tree (DT), k-nearest neighbors (kNN), support vector machine (SVM), or convolutional neural network (CNN) that can be optimized for embedded hardware with limited memory capacity.^17^^,^^18^^,^^19^^,^^20^ Optimization techniques (quantization and pruning) also enable AI models to infer directly on small microcontrollers (mini-MCUs). By running directly on MCUs, wearable devices do not send raw data from soft sensors but instead prioritize edge inference. This helps enhance user security and privacy better than traditional IoT systems.

In a recent study, the group of Ali^21^ developed a fabric-based pressure sensor matrix integrated into the surface of a soft toy to recognize control actions or human-machine interface (HMI) interactions. Through RF and kNN models, the system achieves an accuracy of approximately 83% in real-time control. However, the data were transmitted and processed on a mainframe computer, and the Bluetooth Low Energy (BLE) connection can only transmit data over short distances. In another study, Yang et al.^22^ presented a multifunctional wearable MXene/polydopamine (PDA)@cotton fabric pressure sensor. The sensor demonstrated a working range of up to 146 kPa, with a highest sensitivity of 0.95 kPa^−1^ and a durability of over 3,000 cycles. In the finger motion tracking application, the authors used a deep learning model based on a neural network to classify data collected from a glove. The accuracy of the device reached 87%. However, the work focuses mainly on material improvement. It lacks real-time wireless tracking, and the model does not run on embedded hardware, increasing the risk of data leakage. In one study, Wang et al.^23^ developed a smart shoe insole that integrates soft pressure sensors to monitor the body’s gait. The carbon nanotubes/acetone/polydimethylsiloxane (CNT/ACET/PDMS) structure of the sensor shows a sensitivity of 0.36 kPa^−1^ and a working range of 0–225 kPa. The SVM, CNN, and RF models all achieve a high accuracy of up to 99%. However, according to the system structure, the collected data are transmitted directly to the smartphone via Bluetooth. In-depth analysis and processing are not performed at the embedded board, leading to energy/transmission bandwidth consumption, slow processing/inference, and information security risks.

Based on the aforementioned analysis, the study proposes a fully integrated soft sensor-tiny edge ML co-design framework for small wearable systems in IoT networks. Unlike prior works that either concentrate primarily on material optimization without embedded intelligence^24^^,^^25^^,^^26^^,^^27^ or deploy machine learning models on external/cloud platforms,^28^^,^^29^^,^^30^^,^^31^^,^^32^^,^^33^ this study demonstrates real-time inference on a resource-constrained ESP32S3 MCU. Specifically, the smart interactive glove integrates a CNT-coated elastic fabric pressure sensor with lightweight RF and kNN models executed entirely on-device. The sensor adopts a simple three-layer structure (CNT-fabric/Au electrode/polyimide), achieving good sensitivity, a fast response/recovery, and a compact sensing area (0.5 cm^2^) suitable for fingertip integration. The embedded RF/kNN models are carefully optimized to balance accuracy and hardware constraints. The models achieve classification accuracies of 92.1% and 96.55%, respectively, while occupying less than 750 KB of flash memory and exhibiting extremely low inference latency (0.185/4 ms). In contrast to many soft wearable-AI studies that omit quantitative deployment metrics, the work explicitly reports flash/dynamic random access memory (DRAM) usage, inference time, and real operating power consumption, thereby providing a transparent evaluation of edge feasibility. Importantly, all raw and preprocessed sensor signals remain on the embedded board, and only the final classification labels are transmitted to the cloud via Wi-Fi. The architecture reduces communication overhead and lowers the risk of data leakage, a common issue in cloud-based systems. Users can still monitor the results remotely through internet-connected devices (e.g., smartphones or computers) without exposing raw physiological data. The work builds on our previous CNT-fabric pressure sensor platform,^34^ in which material parameters, including CNT concentration, dipping conditions, and electrode design, were carefully optimized for sensitivity, stability, and durability. This work focuses on system-level integration rather than material innovation. Compared to the previous work that focused on physiological signal monitoring using a tinyCNN model, the present study extends the system in several directions. The application is expanded from health monitoring to low-latency human-machine interaction through a fingertip-integrated smart glove. The deep learning model is replaced with a lightweight feature-based pipeline (RF/kNN), which reduces inference latency from about 180 to 0.185–4 ms. In addition, the sensor design is refined for fingertip-level integration, achieving an enhanced sensitivity (up to 0.46 kPa^−1^). More importantly, the system architecture is redesigned. The new framework integrates multi-channel sensing, sliding-window preprocessing, feature extraction, and on-device inference into a single pipeline. All components are optimized for resource-constrained hardware. In addition, a privacy-aware edge IoT strategy is adopted. All raw data are processed locally, and only prediction labels are transmitted. These changes move the system from a task-specific prototype to a more practical and deployable intelligent wearable platform.

The main contributions can be summarized as follows:

• thin, good-sensitivity fabric pressure sensor for fingertip integration;
• lightweight RF/kNN models on ESP32-S3 with low memory use and ms-level latency;
• measured deployment metrics: flash/DRAM usage and power consumption;
• secure edge IoT: on-device inference and only prediction labels sent to the cloud.

### Flexible sensors and edge IoT-wearable system design

#### Sensor fabrication

The sensor was fabricated using polyimide substrates coated with a thin Au layer, purchased from Thien Lam Electronic Company. 3M thin single-sided adhesive was purchased from 3M Company, Minnesota, United States. The elastic knitted fabric, conductive thread, and 1 wt % CNT ink were prepared from CloApha Company, Seoul, South Korea. The knitted fabric was made of small-sized polyester microfibers intertwined together in a loop structure, which gives the fabric the ability to withstand deformation in all directions and provides high elasticity. The CNT ink concentration and fabric substrate used in the work were inherited from the previously published study,^34^ in which the effects of material composition and dipping conditions on conductive network formation and cyclic stability were systematically investigated. The selected parameters demonstrated stable percolation behavior and low hysteresis under repeated deformation.

**Figure 1:**
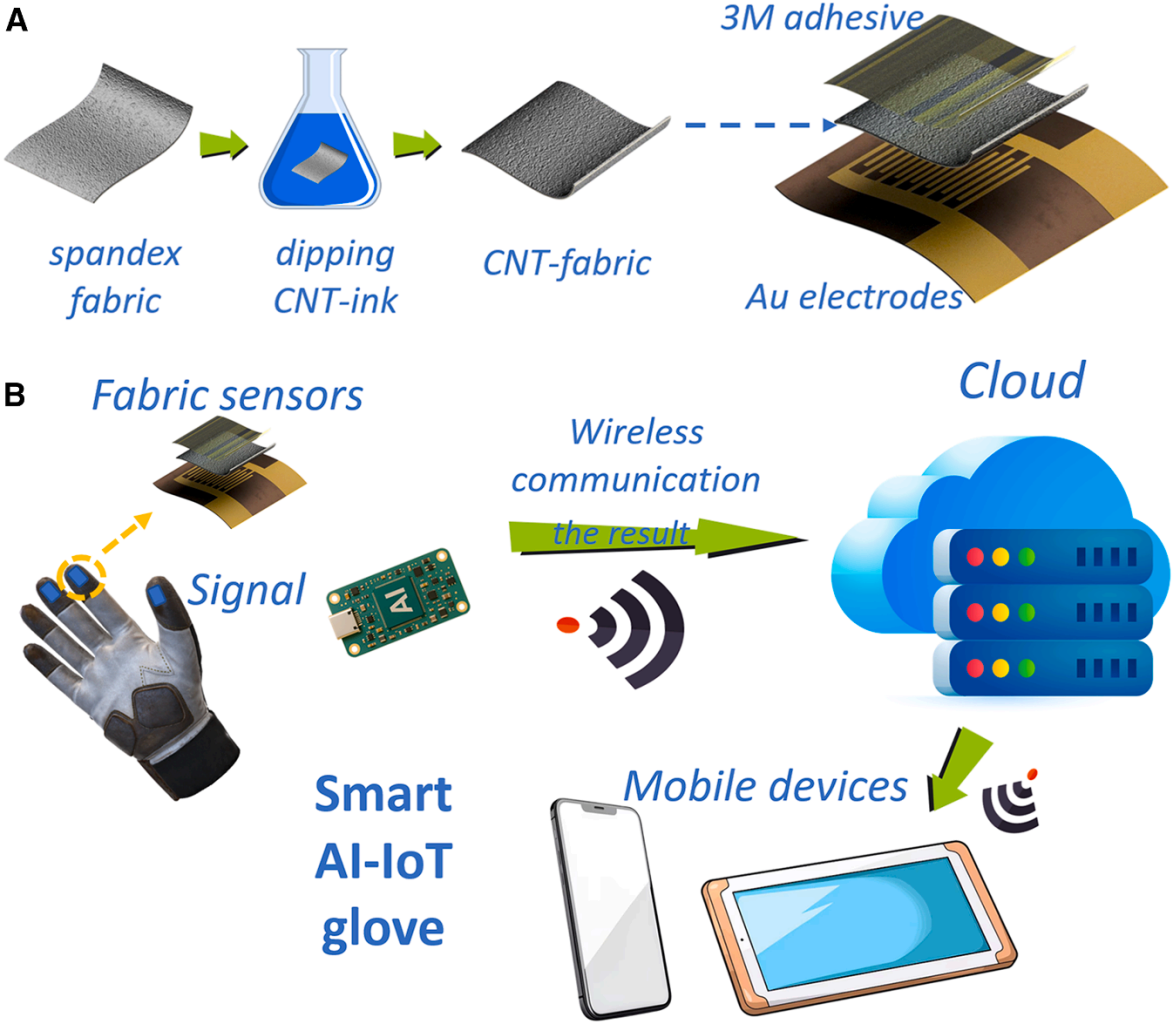
Integrated system (A) Sensor fabrication. (B) Architecture of smart AI-IoT glove.

The electrode structure was designed and printed on the surface of the Au/polyimide layer. The elastic fabric was dipped in CNT ink for 5 min and then dried for 15 min at 120°C to remove excess water (Figure 1A). After drying, the CNT fabric became conductive and had the property of changing resistance under mechanical deformation. Then, the sensor layers were assembled together according to the pre-designed structure. The top layer of 3M single-sided adhesive fixed the position and protected the internal components. Conductive thread was sewn on the electrodes to connect the sensor and the embedded board. A part of the conductive thread was fixed at one point on the electrode with instant adhesive. The method of making connections not only ensures simplicity in construction but also ensures the ability to transmit signals to the main MCU. The interdigitated electrode configuration was also adapted from prior work,^34^ in which the electrode geometry fabricated on a flexible pyralux film via wet etching was experimentally characterized for contact resistance modulation and mechanical stability. The architecture enhances pressure-dependent contact variation between CNT-coated fibers and copper electrodes, thereby contributing to suitable sensitivity and stable operation.

#### Structure of flexible edge-IoT wearable system

The fabric sensor-tiny edge AI co-design architecture describes an approach that takes both the soft sensor and the tiny machine learning model running on the small hardware platform of edge IoT devices (Figure 1B). The data from the soft pressure sensor are usually in the form of a time series. The data are analyzed and pre-processed directly on the embedded board. Features are extracted in both the frequency and time domains and considered based on the collected data information. The most basic features will be used for the tiny machine learning model to make decisions, classify, or predict. Only the final prediction results are transmitted to the server via Wi-Fi connection. Such a design model revolves around the electromechanical characteristics of the sensor, the embedded machine learning model architecture, and the MCU constraints (RAM/flash, cycles, and power).

#### Embedded board and tiny ML algorithms

The embedded board used is XIAO ESP32S3, purchased from Seeed Technology Company, which is small in size (21 × 17.8 mm) with an integrated antenna and a 2.4 GHz Wi-Fi/BLE 5.0 module. The XIAO ESP32S3 provides 8 MB of pseudo-static random access memory (PSRAM) and 8 MB of flash memory. These parameters demonstrate the ability to embed a small-sized AI model while maintaining wireless connections, low power consumption, and a minimal physical size, facilitating easy integration into gloves. RF and kNN were selected for their low computational complexity and suitability for low-dimensional handcrafted features extracted from sliding windows. Compared to deep neural networks, these classical models require fewer parameters and offer deterministic inference latency, which is advantageous for resource-constrained MCUs such as the ESP32. The design platform for RF/kNN is TensorFlow Lite Micro. The selected model uses the features and runs inference directly on the embedded board.

RF is a machine learning algorithm belonging to the ensemble learning group, commonly used for classification tasks. RF builds different DTs on a randomly selected dataset (bootstrap sampling). Each tree will give a result when predicting. The majority of the votes determines the final result, as shown in Equation 1, where C is the set of classes, *I*() is an indicator function, equal to 1 if true, 0 otherwise, and *T*_*b*_(*x*) is the label that tree *b* predicts for sample *x*. RF is effective for complex problems, helps reduce overfitting errors, and is capable of assessing the importance of features.

(Equation 1) $$\hat{y}=\arg\;max_{c\in C}\sum_{b=1}^{B}I\left(T_{b}\left(x\right)=c\right)\text{.}$$

kNN is a robust classification algorithm. Given a new data point, kNN calculates the distance from that point to all points in the training set. The prediction is based on the majority vote from the kNNs. The distance is usually calculated using the Euclidean measure in Equation 2, where *x* = (*x*_1_, *x*_2_, …, *x*_*n*_) is the feature vector of the point to be classified, and *y* = (*y*_1_, *y*_2_, …, *y*_*n*_) is the feature vector of the point in the training set.

(Equation 2) $$d\left(x,y\right)=\sqrt{\sum_{i=1}^{n}{\left(x_{i}-y_{i}\right)}^{2}.}$$

Once the kNNs are found, the predicted label is the one that appears most often, as described in Equation 3, where C is the set of classes, *I*() is an indicator function, equal to 1 if true, 0 otherwise, and *y*_*i*_ is the label of *i*^*th*^ neighbor. With kNN, the system needs to store data samples on the device to calculate the distance. This can be resource-intensive, but it also helps to minimize the inference time (in the case of simple applications and few samples).

(Equation 3) $$\hat{y}=\arg\;max_{c\in C}\sum_{i=1}^{K}I\left(y_{i}=c\right)\text{.}$$

## Results and discussion

### Sensor characteristics

**Figure 2:**
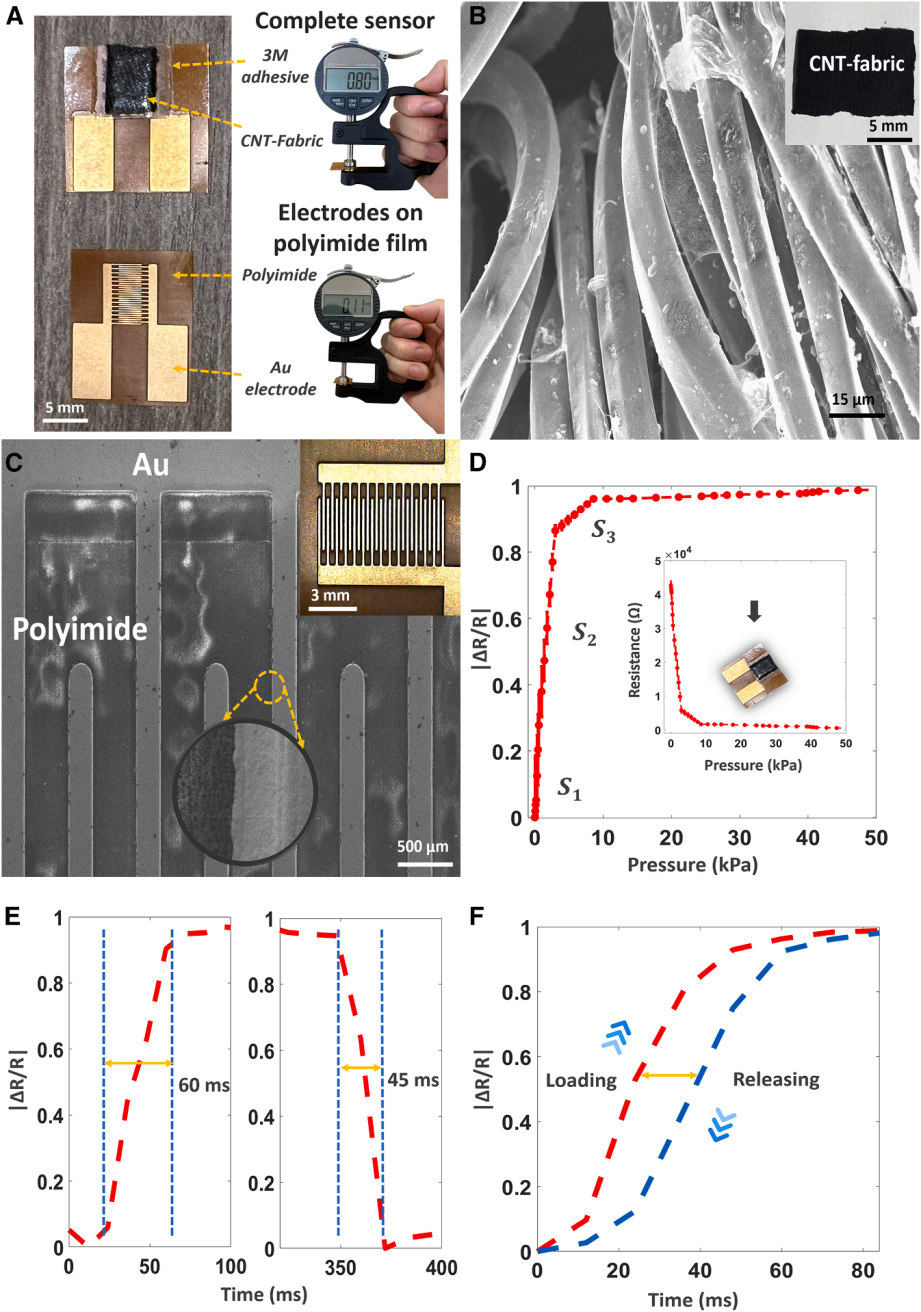
Sensor characteristics (A) Actual image of the electrode layer and complete sensor (scale bars: 5 mm). (B) SEM image of the conductive CNT fabric (scale bars: 15 μm). (C) SEM image of the Au electrodes (scale bars: 500 μm). (D) Change in resistance of the sensor under applied pressure. (E) Response time and recovery time of the sensor. (F) Hysteresis when loading and releasing.

The structure and parameters of the fabricated soft pressure sensor are exhibited in Figure 2. Figure 2A shows the main 3-layer structure. Two Au electrodes are shaped on a polyimide substrate. The thickness of the layer is about 0.11 mm. Above is the CNT fabric layer. On top is the 3M single-sided adhesive layer. The microscopic image of the CNT conductive fabric is shown in Figure 2B. The elastic fabric is made of bundles of fibers knitted together. The size of each fiber is about 10 μm. The CNT conductive particles adhere to the surface of the fibers, and the fiber bundles give the fabric the ability to conduct electricity. However, due to the weak adhesion, the CNT particles can fall off the fibers, gradually reducing the conductivity after many deformations. In Figure 2C, the comb-shaped interwoven Au electrode structure has a single line length of about 5 mm and a width of about 0.2 mm. The distance between two single lines is 0.4 mm. Four electrode spacings are evaluated (Figure S1). Figure S1 shows the interdigitated comb-shaped Au electrode structure, where each electrode line has a length of ∼5 mm and a width of ∼0.2 mm. The spacing between adjacent interdigitated fingers in samples S0–S3 is gradually increased from 0.4 to 0.6, 0.8, and 1.0 mm, respectively, with an effective sensing area of approximately 5 × 10 mm^2^. All samples exhibit a rapid increase in resistance change at low pressures (<∼5 kPa), followed by a gradual saturation at higher pressures. Among them, S0 shows the highest response across the entire pressure range, approaching ∼1.0 above ∼10 kPa, while S1, S2, and S3 reach slightly lower values of ∼0.98, ∼0.94, and ∼0.90, respectively. The enhanced performance of S0 is attributed to the smaller electrode spacing (0.4 mm), which strengthens the modulation of conductive pathways under compression. Therefore, the S0 configuration was selected for subsequent device fabrication. The sensing area is about 5 × 10 mm^2^. In a small area, the more interwoven electrode lines there are, the better the sensing ability of the sensor is. The overall thickness of the entire structure is about 0.8 mm. The small size makes it easy to integrate the sensor into many different locations or spaces in practical applications, such as fingertips or wrist joints.

The sensing mechanism is governed by pressure-induced modulation of the percolation network formed by CNT-coated fibers in contact with interdigitated electrodes (Figure S2). The structure of knitted fabrics plays an important role in determining the performance of pressure sensors. The fabric consists of numerous microfibers twisted into yarn bundles, which are further knitted to form the overall structure. This configuration provides excellent flexibility, deformation tolerance, and reliable repeatability. When pressure is applied to the knitted fabric, the electrical conductivity increases due to the compression of the yarn loops. This compression reduces the distance between conductive particles (CNTs), thereby facilitating electron transport between the two electrodes. In other words, the looped architecture of the weft-knitted structure allows substantial deformation under external pressure, leading to variations in the resistance of the conductive fiber network. As a result, this structural feature enhances the pressure sensitivity and recovery behavior of the CNT-based fabric sensor, ultimately improving its overall sensing performance. Figure 2D illustrates the change in resistance under the action of compressive pressure. Five independently fabricated sensors were characterized under identical experimental conditions. The reported sensing performance values represent the mean response, and the error bars indicate the standard deviation between devices. The variation is negligible. The low standard deviation indicates good reproducibility and reliability. Sensitivity (*S*) is one of the most important parameters to evaluate the performance of a flexible pressure sensor. As described in Equation 4, *S* is usually defined as the ratio between the change in resistance signal and the change in applied pressure, where *R*_0_ is the initial resistance value, Δ*R* is the change in resistance, and Δ*P* is the corresponding pressure change.

(Equation 4) $$S=\frac{\Delta R/R_{0}}{\Delta P}\text{.}$$

The sensor displays a good sensitivity of *S*_1_ = 0.46 kPa^−1^ in the pressure range of 0–1 kPa, *S*_2_ = 0.29 kPa^−1^ in the pressure range of 1–5 kPa, *S*_3_ = 0.11 kPa^−1^ in the pressure range of 5–10 kPa, and a moderate sensitivity of *S*_4_ = 0.04 kPa^−1^ in the pressure range of 10–50 kPa. When the pressure level exceeds 50 kPa, the change in resistance becomes less and is not suitable for use. To evaluate the capability of detecting weak pressure stimuli, the limit of detection (LOD) was estimated based on the signal-to-noise criterion using the relation (Equation 5):

(Equation 5) $$LOD=\frac{3\sigma_{noise}}{S}\text{,}$$

where *σ*_*noise*_ represents the standard deviation of the baseline signal under zero applied pressure, and *S* is the sensitivity in the low-pressure regime. The baseline noise was recorded under no-load conditions for 30 s, and the corresponding standard deviation was extracted from the steady-state signal. Based on the measured noise level and the sensitivity in the linear region, the calculated LOD is approximately 15 Pa, indicating that the sensor can detect subtle pressure variations relevant to wearable physiological monitoring. Response time is the time it takes after pressure is applied until the sensor signal reaches a certain percentage of its maximum value (usually 90%). The value indicates the ability to respond quickly to deformations. Conversely, recovery time is the time it takes for the sensor signal to return to its original state after the deformation is removed. The value reflects the speed at which the sensor returns to its resting state. Figure 2E shows the response time and the recovery time to the increase and removal of pressure. These values are 60 and 45 ms, respectively. These fast response times highlight the potential of the sensor for use in most applications. Another parameter is a small hysteresis, which is depicted in Figure 2F. The hysteresis represents the difference in the respective resistance values during the loading and releasing cycles; the smaller the difference, the better the sensor.

**Figure 3:**
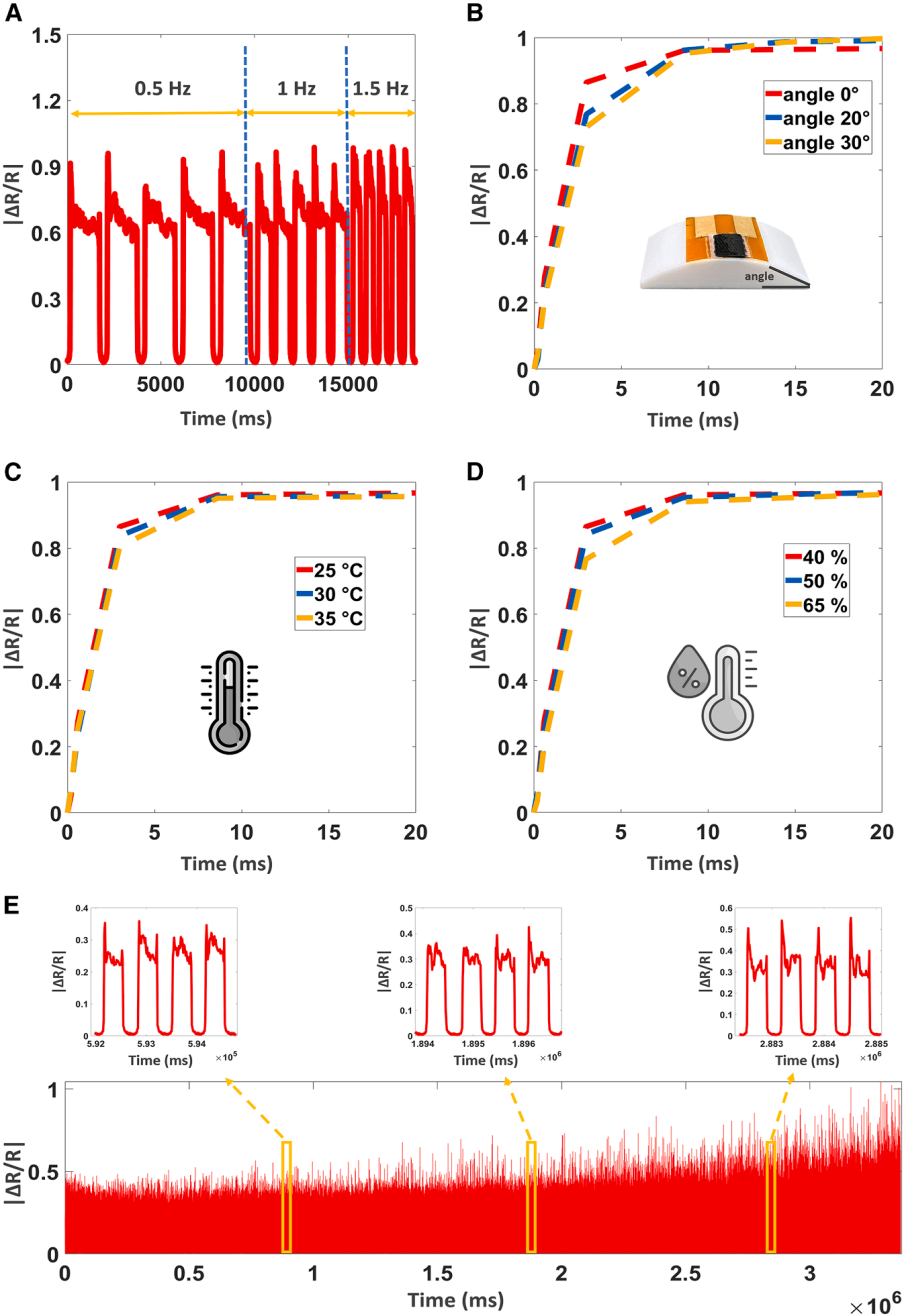
Sensor characteristics (A) Response of the sensor to different frequencies of applied force. (B) Time-domain response under different bending angles. (C) Time-domain response under stepwise applied pressures at different temperatures. (D) Time-domain response under stepwise applied pressures at different humidities. (E) Durability of the sensor during 5,000 loading/releasing cycles.

The stability under different frequencies of force is tested in Figure 3A. At all three loading frequencies (0.5, 1, and 1.5 Hz), the shapes of the response curves demonstrate consistency. One of the outstanding features of soft sensors is their ability to function effectively on uneven surfaces, which is particularly suitable for many practical requirements. To verify this, the sensor was placed on three different curved surfaces (0°, 20°, and 30°) created by a 3D printer. Figure 3B illustrates a small resistance change in the sensor on the curved surfaces. Figures 3C and 3D and Figure S3 show the resistive response of the fabric pressure sensor under different temperatures (25°C, 30°C, and 35°C) and relative humidity levels (40%, 50%, and 65%). Within the room-temperature range, the sensor shows negligible variation in resistance and pressure response. In contrast, increasing humidity causes a slight decrease in sensor sensitivity. The effect is mainly attributed to moisture absorption by the fabric fibers and the CNT conductive network, which alters the contact resistance. Despite the influence, the sensor maintains stable and reliable performance under typical indoor environmental conditions. The environmental tests were limited to a moderate indoor range (25°C–35°C and 40%–65% relative humidity), which reflects typical wearable-use conditions. The experiments focused on baseline operational stability, not full robustness under extreme environments. Broader testing over wider temperature and humidity ranges, outdoor conditions, sweat exposure, and long-term storage is needed to better assess practical durability and support material or packaging optimization. Further improvements may include moisture-resistant materials and protective encapsulation layers. Temperature and humidity compensation can also be applied to reduce signal drift. Figure 3E shows the change in sensor resistance after 5,000 loading/releasing cycles at 15 kPa and 2.5 Hz. The signals collected from the 1,200th, 2,400th, and 3,600th cycles exhibit similar patterns, indicating stable operation during approximately the first 3,500 cycles. Beyond this point, the maximum resistance gradually increases, with a total variation of up to ∼20% in the final cycles. In the durability test, cyclic compressive loading was applied to reflect the intended use in fingertip pressure sensing during grasping. The observed degradation is mainly attributed to the weak physical adhesion of CNT particles to the textile microfibers after dip-coating and drying. Under repeated compression and friction between yarn bundles, parts of the CNT network progressively detach from the fiber surface, leading to increased resistance and signal drift, as supported by the SEM observations in Figure S4. At 0 cycles (Figure S4A), the CNT layer forms a relatively continuous coating on the fiber surface, with noticeable CNT aggregates distributed along the fiber. After 5,000 cycles (Figure S4B), partial removal of these aggregates can be observed, and the CNT coverage becomes less uniform, indicating the onset of mechanical degradation under repeated deformation. When the number of cycles increases to 10,000 cycles (Figure S4C), the fiber surface appears smoother, and the amount of visible CNT clusters further decreases, suggesting that some CNTs have gradually detached from the substrate during cyclic loading. Although the large-scale morphology of the fibers remains intact, the progressive reduction of CNT coverage and surface roughness indicates partial CNT detachment, which is consistent with the observed decline in sensor performance after prolonged use. Post-cycling SEM images are used as qualitative evidence of the proposed degradation mechanism. Quantitative analysis of CNT coverage, surface fraction, or mass retention is beyond the scope of this study. However, the combination of morphological observation and electrical drift supports a consistent interpretation of degradation. More rigorous quantitative methods, such as image-based analysis or spectroscopy, will be addressed in future work. For further improvement, stronger CNT adhesion could be achieved through approaches such as polymer-binder-assisted CNT inks, surface functionalization of the textile substrate, repeated infiltration/drying, or encapsulation/interlocking layers to reduce particle loss during cyclic deformation. The cyclic test evaluates sensor repeatability under controlled loading but does not fully reflect real-world conditions. Thus, it indicates initial mechanical reliability rather than full lifetime performance. The classification pipeline remains robust to moderate signal degradation because it relies on normalized temporal features. To mitigate the impact of the long-term drift, a preprocessing pipeline based on a sliding-window strategy was implemented, enabling stable inference despite material degradation. The raw resistance signal *R*(*t*) was sampled at 100 Hz and segmented into 2.5 s windows (250 samples per window). Within each window, a moving mean filter was first applied to suppress high-frequency noise, yielding the filtered signal $\tilde{R}\left(t\right)$. Subsequently, min-max normalization was performed locally within each window according to the following equation:

(Equation 6) $$R_{norm}=\frac{\tilde{R}\left(t\right)-R_{\min}}{R_{\max}-R_{\min}}\text{,}$$

where *R*_*min*_ and *R*_*max*_ denote the minimum and maximum values computed within the current window. The adaptive normalization prevents the accumulation of baseline drift and reduces dependence on absolute signal amplitude.

While CNT-based fabric sensors have been widely reported, the contribution of the present sensor lies in its integration-oriented optimization. The sensing layer, electrode geometry, and small thickness were engineered to enable stable fingertip-level deployment with low hysteresis, rapid response, and a low LOD. In addition, durability up to 3,500 cycles under repeated loading ensures compatibility with low-latency wearable interaction tasks. Rather than proposing a new sensing material, the work demonstrates how a previously validated CNT-fabric platform can be configured and quantified for reliable embedded AI integration. Notably, the classification framework relies primarily on relative temporal features extracted from normalized windows rather than absolute resistance magnitudes. Therefore, gradual amplitude attenuation due to CNT network rearrangement has limited impact on feature separability. Experimental validation further shows that the classification accuracy decreases only slightly from 96.55% (initial state) to 94.80% after 3,500 loading cycles, confirming the robustness of the system against long-term degradation. Detailed performance evaluation is provided in real-time object classification.

### Intelligent glove-integrated flexible sensors

**Figure 4:**
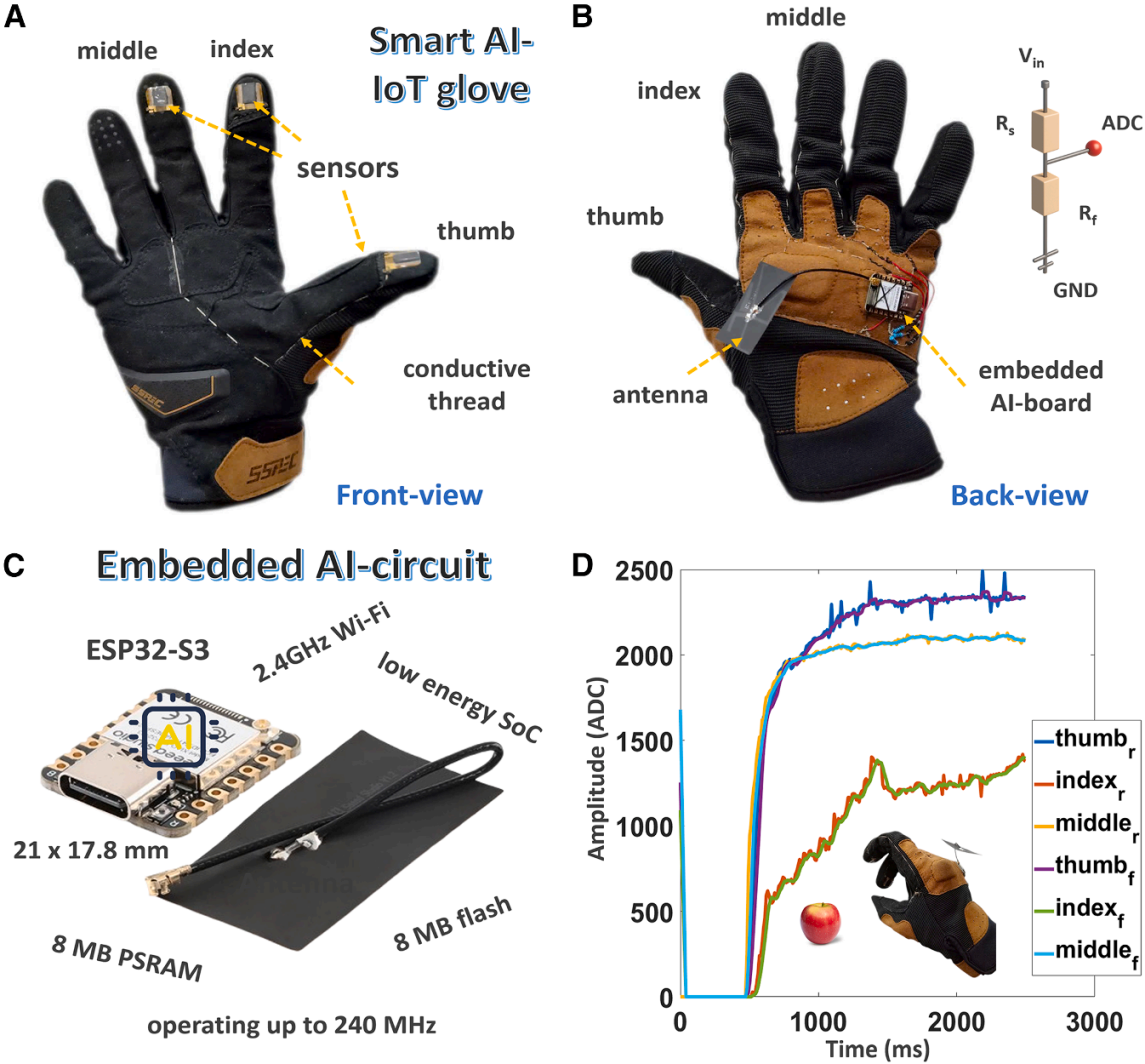
Intelligent glove-integrated flexible sensors and tiny ML models (A) Front structure of the glove. (B) Back structure of the glove. (C) ESP32S3 central embedded board for tiny ML models. (D) Raw and filtered signals collected from three sensor channels on the glove.

The IoT-wearable device consists of several components: soft sensors, connectors, a small main board, small ML models, and an antenna. All components are mounted on a complete glove. Figure 4A shows the front of the glove. Three soft sensors are mounted on the fingertips of the thumb, index, and middle fingers. The thumb, index, and middle fingers were selected because they play a primary role in both precision and power grasps during daily object manipulation. From a biomechanical perspective, these digits generate the dominant contact forces. They also encode most of the pressure patterns associated with object shape, stiffness, and weight distribution. From an embedded system perspective, limiting the sensing array to three channels provides a favorable trade-off between information content and hardware simplicity. The design reduces the number of conductive interconnections, analog acquisition channels, and extracted features. As a result, it lowers the memory footprint and computational load of the TinyML pipeline while preserving sufficient information for reliable classification. The design choice is motivated by both human grasping behavior and practical edge-device constraints. The sensors are small in size, so they fit within the fingertip area. Figure 4B displays the back of the glove with an AI-embedded board. We use conductive threads to connect the two electrodes of each sensor to the processing circuit. The conductive threads are directly sewn to the electrodes and the pin holes on the board, eliminating the need for soldering. In Figure 4C, the board module is small (21 × 17.8 mm), has 8 MB of memory, and includes a built-in 2.4 GHz Wi-Fi module. The ESP32 platform supports integration of ML models through libraries (like TensorFlow Lite or Edge Impulse). In the aforementioned structure, all components (except the main board) are flexible soft materials. The research goal is to develop an entirely flexible system that maximizes comfort for the wearer. Due to the limitations of the technology, the central processing board is still a hard component. Thanks to its small size and light weight, the hard component will not affect the wearer when performing specific operations. The analog-to-digital converter (ADC) converts continuous analog signals from sensors into digital signals that the computer can use. The reason why the ADC is necessary is that most advanced calculations use digital values. The ADC value is calculated based on a voltage divider circuit according to Equation 7, where *V*_*in*_ is the analog input voltage, *V*_*ref*_ is the reference voltage, and *n* is the number of ADC bits. The ESP32S3 has a default resolution of 12 bits. The voltage divider circuit was implemented using precision through-hole resistors. Although such components typically exhibit tolerance in the range of ±1%–5%, their influence on system performance is minimal in the present design. Since the classification framework relies on normalized sliding-window signals and relative temporal features rather than absolute voltage magnitudes, static offset or scaling variations introduced by resistor tolerance do not significantly affect inference accuracy. In addition, the signal acquisition window (2.5 s) is relatively short, and measurements were conducted under stable indoor temperature conditions, thereby minimizing thermal drift. While the current prototype is not fabricated on a dedicated printed circuit board (PCB), the short signal routing and filtering strategy ensures stable operation, as validated by the consistent classification accuracy reported in the following section. Figure 4D describes that the signal collected from the soft pressure sensor is in a time-series form. The signals have been filtered and smoothed with a mean filter. The principle of the mean filter is to replace the value at a point by the average of the neighboring points in a given sliding window. The method helps to remove noise and is easy to implement.

(Equation 7) $$ADC_{value}=\frac{V_{in}\times 2^{n}}{V_{ref}}\text{.}$$

### Real-time object classification

**Figure 5:**
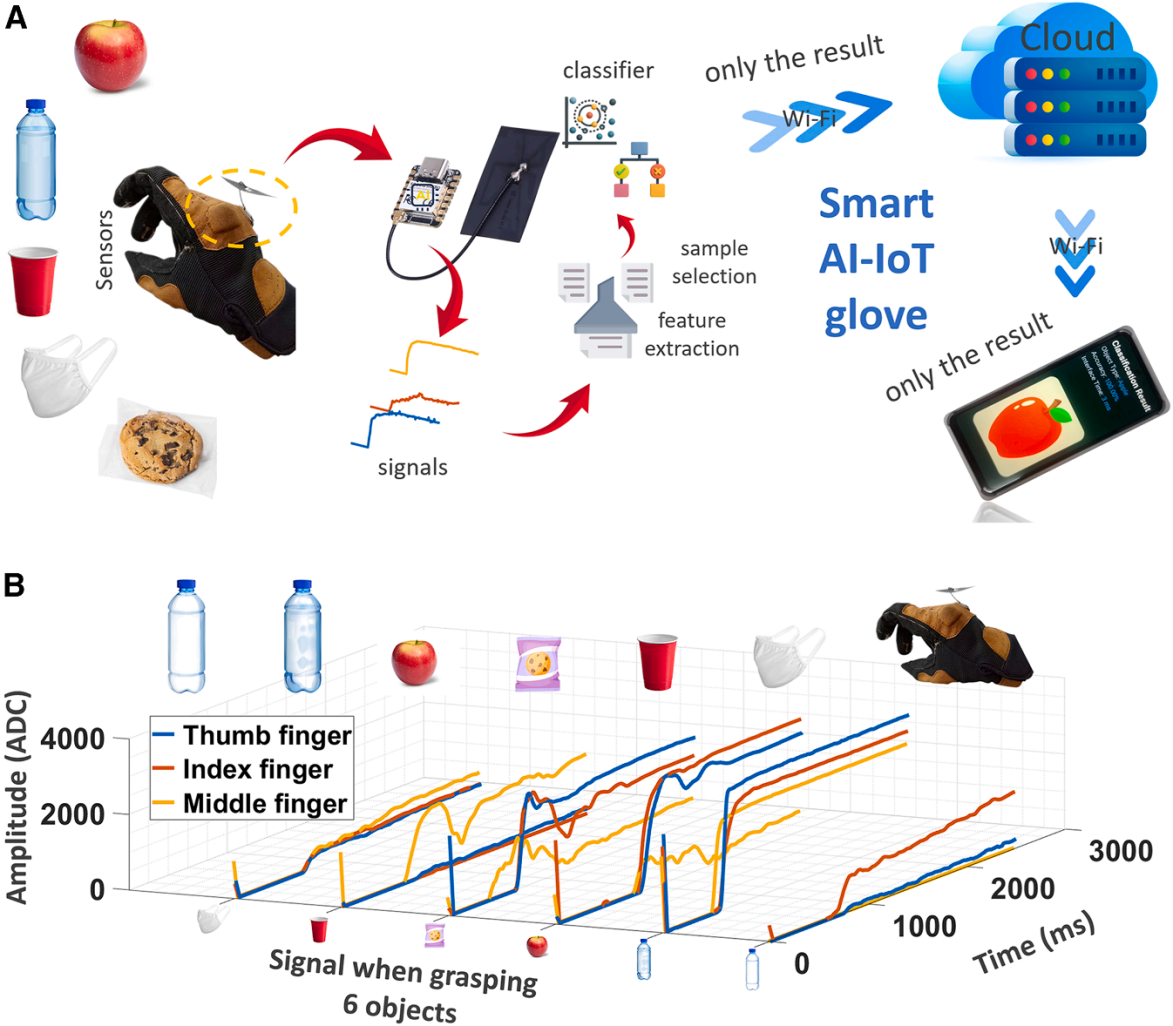
Intelligent IoT-wearable system (A) Operational structure of the entire system. (B) Signal patterns corresponding to the actions of holding different objects.

To demonstrate the on-device monitoring capability, several representative objects were prepared, including an empty plastic bottle, a water-filled plastic bottle, an apple, a plastic cup, a bag of cookies, and a cloth mask. The demonstrated six classes are an experimental setting, while the practical class capacity depends on feature separability and embedded resource constraints. Figure 5A illustrates the working process of the flexible intelligent IoT system. Data were collected from three healthy male participants aged 23–35 years. All participants were right-handed and had no reported hand-related disorders. Each participant performed grasping actions for six object categories. The order of object presentation was randomized to avoid sequence bias, and the number of samples was evenly distributed among participants and object classes. The object classification dataset was collected under controlled indoor conditions, so it does not fully represent real-world user diversity. Differences in gender, age, finger size, hand biomechanics, skin condition, and grasping habits may affect pressure distribution and feature patterns. Therefore, the results should be a proof-of-concept validation of the proposed integrated sensing and edge-inference framework. In addition, the wearability of the gloves was assessed through typical gripping tasks with and without gloves (20 trials per task under each condition). Task completion time, success rate, number of failed grasps, and a range-of-motion (ROM) proxy were quantified. Table S1 shows that the glove introduces only minimal performance degradation. The completion time increased slightly (6%–13%), while the success rate remained at or above 95% across tasks. The ROM proxy remained above 95%, indicating negligible restriction in finger movement.

Signal acquisition was triggered when the sensor output exceeded a predefined threshold, indicating contact onset. Each grasping trial was then recorded for 2.5 s at a sampling frequency of 100 Hz (250 samples per window), with participants allowed to grasp objects naturally to reflect realistic usage conditions and improve dataset robustness. The 2.5 s acquisition window was chosen as a compromise between signal completeness and low-latency responsiveness. During grasping, pressure signals from the three fingertip channels evolve through a short transient phase before stabilizing; shorter windows may truncate these characteristic patterns, whereas longer windows would increase latency without providing significant additional information. Since contact detection (∼60 ms) and model inference are fast (as shown below), the acquisition window dominates the overall delay, making 2.5 s a practical balance between accuracy and on-device performance. The corresponding signal patterns from the three fingertip sensing channels are shown in Figure 5B. A total dataset of approximately 1,700 samples was used, of which 1,390 were experimentally collected and the remainder were generated via data augmentation. The dataset was evenly distributed among the six object categories, with 60% used for training and 40% for testing.

**Figure 6:**
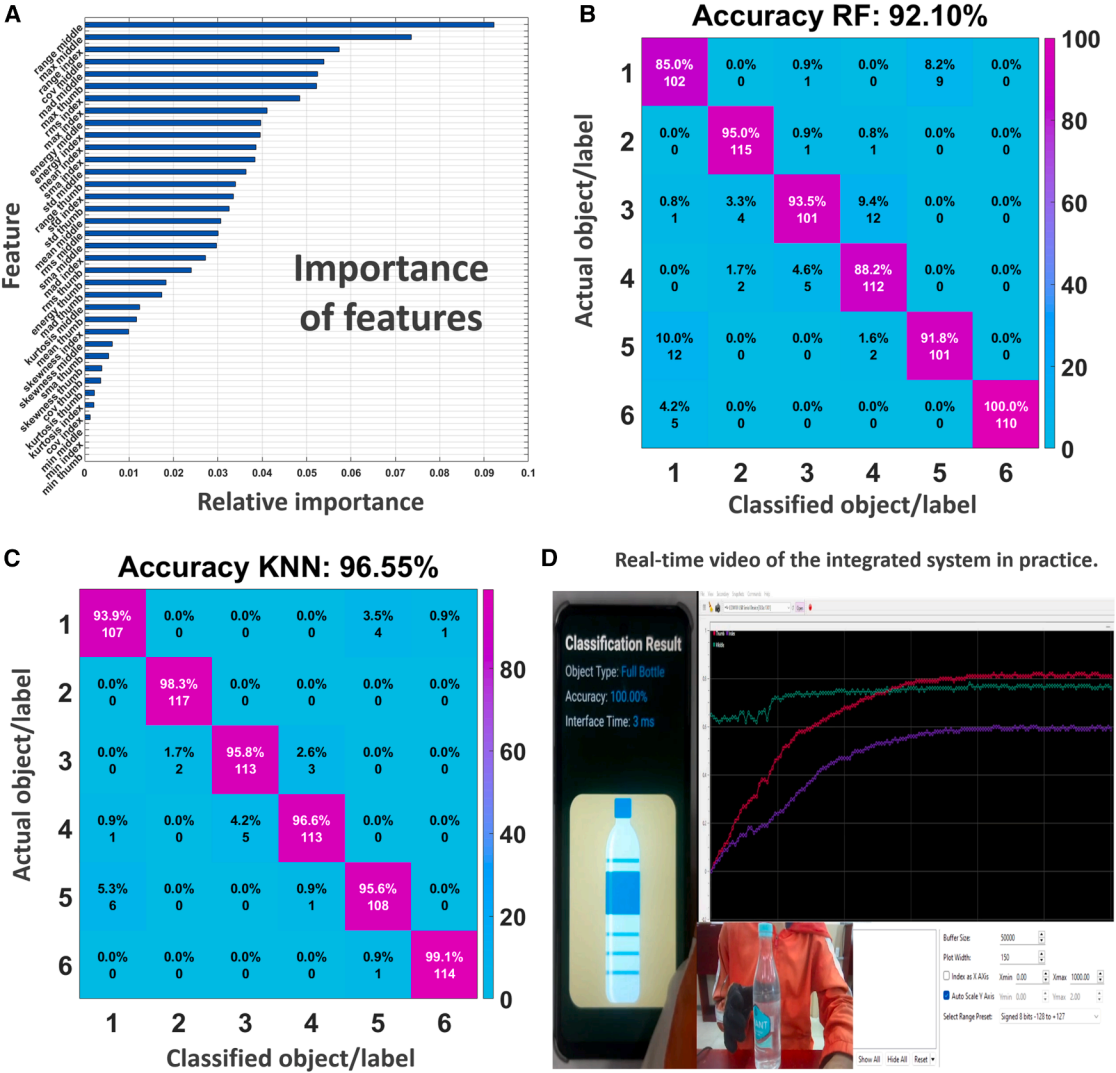
Tiny machine learning models (A) Importance of features. (B) Confusion matrix of RF model on the test set. (C) Confusion matrix of kNN on the test set. (D) Real-time video of the integrated system in practice.

The two ML models used in the study are RF and kNN. For RF, the model parameters are pre-trained on TensorFlow. The model is then packaged and uploaded to the ESP32 via TensorFlow Lite Micro. By analyzing the time and frequency domains, we obtained 12 features for the RF model, including max, sma, std, rms, min, cov, mad, mean, energy, range, skewness, and kurtosis. Here, mean represents the average value, std represents the standard deviation, max is the largest value, min is the smallest value, range is the range of values, energy represents the energy level, mad is the mean absolute deviation, sma represents the signal magnitude area, skewness represents the deviation of the signal in the frequency domain, kurtosis is the sharpness of the signal in the frequency domain, rms is the root-mean-square value, and cov is the relative variability of the signal. Each sensor channel has 12 features, so there are a total of 36 features for all three sensors. Figure 6A visualizes the influence of the features. The importance of the features is evident in their influence on the final inference result. The representation is usually unitless. The sum of the importance of the features is 1. Table 1 shows the parameters of the RF model. The parameter set is designed to balance accuracy and generalization. The number of DTs is 50, which is stable enough and not too computationally intensive. RF uses “entropy” to evaluate the splits and “sqrt” to select features at each split, thereby reducing overfitting. The depth limit is 10, and pruning with ccp alpha is 0.0015 to avoid overly complex trees. Bootstrap sampling and a “max samples” value of 0.7 are used to increase randomness and reduce variance.

**Table 1:** Parameters of the RF model

Parameter	Description
n_estimators_	50
criterion	“entropy”
max features	“sqrt”
max depth	10
bootstrap	true
max samples	0.7
ccp alpha	0.0015

Figures 6B and 6C display the confusion matrix obtained when testing the models on the dataset. The horizontal row represents the classified object type (results from RF), and the vertical row represents the actual material type. The numbers 1 through 6 represent different types of objects, which are, respectively, an empty bottle (1), a full bottle of water (2), an apple (3), a bag of cookies (4), a cup (5), and a mask (6). The diagonal row (left to right) in the matrix exhibits the correct classification of the held object. The remaining cells show the model’s errors. Accordingly, the RF model achieved an overall accuracy of 92.1% (Figure 6B), with the lowest performance observed for the empty bottle (85%) and the apple (88.2%). The highest accuracy was obtained for the mask (100%). The relatively lower accuracy for the empty bottle is mainly due to its grasping pattern being mechanically similar to those of other lightweight objects, particularly in terms of shape, stiffness, and required gripping force. Compared with the full bottle, it produces weaker and more variable pressure distributions across the three fingertip channels, leading to partial overlap in the feature space and a higher likelihood of misclassification.

Table 2 presents the primary hyperparameters of kNN, including n_neighbors_ set to 3, weights as “uniform,” p as 2, and metric as “minkowski.” The model is using three nearest neighbors, with equal weights, Euclidean distance, and letting sklearn choose how to find neighbors. The parameter structure will be suitable for not-too-noisy data and a moderate size of three sensor channels. For kNN, the most representative samples (50 samples for each label) are stored directly on the processing board as the basis for new data inference. kNN achieved a high overall performance with an accuracy of 96.55% (Figure 6C). When analyzing the signal while holding the empty bottle, the model showed 93.9% accuracy. These values for the apple, the bag of cookies, and the cup are all above 95%. In particular, the model achieved high accuracy in recognizing the full water bottle with 98.3% and the mask with 99.1%. In the present feature-based framework, kNN achieves higher performance with significantly lower computational overhead, demonstrating the efficiency of classical models under structured feature extraction. Although quantized neural networks under the TinyML paradigm are widely used for embedded AI, they are primarily advantageous for raw high-dimensional time-series inputs. In the proposed system, classification relies on structured, handcrafted features, where classical models achieve high accuracy without requiring deep network optimization or complex compression procedures.

**Table 2:** Parameters of the kNN model

Parameter	Description
n_neighbors_	3
Weights	“uniform”
leaf size	30
P	2
metric	“minkowski”
algorithm	“auto”

Table 3 summarizes the deployment characteristics of RF and kNN on the ESP32S3 platform. Although RF performed competitively, kNN achieved higher classification accuracy and was therefore selected for practical deployment. In the resource-constrained setting, classical models are more suitable than deep architectures for three main reasons. First, they require fewer parameters and avoid the additional complexity associated with neural network compression and quantization. Second, they provide deterministic and extremely low-latency inference on embedded hardware such as the ESP32S3. Third, they already achieve high accuracy in our application—92.1% for RF and 96.55% for kNN—while occupying less than 750 KB of flash and requiring only 0.185 and 4 ms inference time, respectively. In contrast, our previous work using a 1D-CNN on raw time-series data achieved 95.2% accuracy, slightly lower than the feature-based kNN model, while incurring significantly higher inference latency (over 100 ms). Therefore, for the present structured-feature wearable scenario, RF and kNN offer a more favorable trade-off between accuracy, complexity, and deployment than deep learning approaches. Model fusion was also not adopted to avoid the increased memory footprint and inference latency associated with storing multiple parameter sets and performing sequential prediction operations.

**Table 3:** Parameters of the RF and kNN models when deployed on embedded hardware

Parameter	RF	kNN
Flash memory	590 KB	740 KB
DRAM	23 KB	32 KB
Accuracy	92.1%	96.55%
Inference time	0.185 ms	4 ms

To ensure a seamless transition from experimental evaluation to real-world application, the complete workflow—from signal acquisition and preprocessing to offline training, parameter export, and on-device inference—was implemented on the ESP32 platform. Given the limited hardware resources typical of IoT devices, integer-based implementation and parameter optimization were applied to reduce memory usage and inference latency. The memory footprint and inference time of both models are summarized in Table 3, demonstrating their suitability for embedded deployment. The operation of the system is demonstrated more specifically and realistically in Figure 6D and Video S1. In addition, power consumption is a factor that cannot be ignored when considering a small IoT device. Equation 8 calculates the power usage of the system, where U is voltage and I is current. Through a multimeter, the glove used an average of 70.3 mA (260.1 mW) when operating at full power and only 59 mA (218.3 mW) when only maintaining a wireless connection. The system uses a 3.7–750 mAh lithium battery as a power source, meaning a total operating time of up to 10.7–12.7 h. The device only uploads the classification label or the final inference result to the cloud via a 2.4 GHz Wi-Fi connection. Users can remotely monitor the results by connecting their devices (phones and computers) to the cloud. The aforementioned design demonstrates both the potential of flexible sensors and the capabilities of embedded AI on small boards. This architecture represents a typical structure for edge AI wearable systems. In addition, Table S2 shows the power consumption under different operating modes. The total power increases from 218 mW in the idle state to about 260 mW in full operation. Sensor acquisition and preprocessing cause a moderate increase due to continuous sampling and feature extraction. The result reflects the model’s low complexity and short latency. Overall, wireless communication and sensing account for most of the power consumption, while on-device AI has a limited impact. These results support the energy efficiency of the proposed system for wearable edge applications.

(Equation 8) $$P_{consumption}=U\times I\text{.}$$

Table 4 summarizes the latest research in the field of smart wearables that integrate soft pressure sensors in recent years. Among them, the studies^24^^,^^25^^,^^26^^,^^27^ focus on developing materials and sensors, without incorporating AI. In this direction, our research creates a sensor with higher sensitivity. In addition, the sensor achieves a small response/recovery time while maintaining a significantly smaller thickness than most papers in the table. We have the wireless communication module (Wi-Fi) to transmit data over long distances, which is especially necessary for mobile wearable applications. The approach^33^ created a fabric sensor-IoT system that transmits data to the cloud via Wi-Fi; however, this introduces a higher risk of data leakage and has not yet integrated AI into the system. Other studies^28^^,^^29^^,^^30^^,^^31^^,^^32^ integrate AI, but all run on large hardware systems (less flexible and not mobile) or on the cloud (prone to data leakage). Those studies also rarely use wireless communication technologies for the device. In our paper, the inference time of the model is clearly exhibited with an extremely fast speed of 0.185/4 ms. Most studies that integrate AI for soft sensors often do not evaluate the value or require a considerable time. Through Table 4, the outstanding points of the proposed system become clearer, confirming its excellent ability in practice.

**Table 4:** Comparison of soft pressure sensor integrated systems in wearable applications

Reference	Sensitivity (kPa^−1^)	Response/release time (ms)	Thickness (mm)	Durability (cycle)	Applied AI	Embedded AI	Inference time (ms)	Wireless connection
Xiao et al.^24^	0.21	300/200	–	5,000	no	–	–	no
Wang et al.^25^	0.1	119/59	>10	1,000	no	–	–	no
Tian et al.^26^	0.0943	100/60	–	4,000	no	–	–	Bluetooth
Yu et al.^27^	0.0128	102/83	22	10,000	no	–	–	–
Xia et al.^28^	6.89	100/110	10	5,000	CNN	no	–	no
Gu et al.^29^	1.6	52/40	0.76	1,000	CNN	no	–	no
Fang et al.^30^	–	100/500	0.9	2,500	SVM	no	–	no
Su et al.^31^	0.398	–	–	5,000	ANN	no	–	no
Zhang et al.^32^	5.35	–	>7.5	1,000	kNN	no	–	radio frequency
Srinivasan et al.^33^	0.47	126/46	0.86	20,000	no	no	–	Wi-Fi
Ours	0.46	60/45	0.8	3,500	RF/kNN	RF/kNN	0.185/4	Wi-Fi

The aforementioned results demonstrate that the sensing material and electrode architecture provide stable and reproducible electrical responses for wearable monitoring. The sensing platform has been previously optimized. Therefore, the study focuses on system-level co-design, including signal conditioning, ADC configuration, noise mitigation, model compression, and deployment on resource-constrained hardware. In addition, the privacy advantage is achieved at the system level rather than through cryptographic protection. Raw multi-channel signals and preprocessed data remain on the glove, as these data may reveal detailed user behavior. Only the final classification label is transmitted. The design limits the transfer of fine-grained interaction data. Compared with cloud-based systems, it reduces data exposure and communication overhead by performing inference on the device. The approach does not replace encryption or secure communication protocols. However, it provides a practical reduction in privacy risk for edge-IoT wearable sensing. The system uses a compact ESP32S3 board, thin fingertip sensors, conductive-thread interconnects, and a simple voltage-divider interface without auxiliary electronics. Thus, the proposed platform is compact and cost-effective. The system-oriented perspective distinguishes the work from conventional material-focused studies and highlights a scalable pathway toward intelligent wearable systems.

The proposed smart glove demonstrates the successful integration of thin fabric pressure sensors and TinyML models into a compact edge-IoT wearable platform. The CNT-fabric pressure sensor exhibited a sensitivity of 0.46 kPa^−1^, fast response/recovery times of 60/45 ms, and stable operation over approximately 3,500 cycles. Integrated with an ESP32S3 MCU, the embedded RF and kNN models achieved classification accuracies of 92.1% and 96.55%, respectively, while maintaining low memory consumption and millisecond-level inference latency. Beyond sensing performance, the work highlights a practical edge-AI architecture for wearable systems. All signal preprocessing and inference are performed locally on the embedded device, and only prediction labels are transmitted through Wi-Fi, reducing communication overhead and limiting exposure of raw user data. The combination of flexible sensing materials, lightweight machine learning models, and on-device intelligence provides a promising framework for future healthcare monitoring, assistive technologies, and human-machine interaction applications.

### Limitations of the study

Several limitations of the present work should be acknowledged. The object-classification dataset was collected from a limited number of participants under controlled indoor conditions. Therefore, the results may not fully represent variations in grasping behavior, hand biomechanics, environmental conditions, and long-term wearable use. The current system recognizes only six representative object classes. Larger and more diverse datasets are still required to evaluate scalability and generalization. Although the CNT-fabric sensor showed stable operation over approximately 3,500 cycles, further improvements in material adhesion and encapsulation are needed for better long-term durability under repeated deformation. In addition, the privacy-aware architecture reduces raw-data transmission through on-device inference, but it does not replace dedicated encryption or secure communication protocols for high-security applications.

## Resource availability

### Lead contact

Further information and requests for resources should be directed to and will be fulfilled by the lead contact, Chi Cuong Vu (cuongvc@hcmute.edu.vn).

### Materials availability

This study did not generate new unique materials.

### Data and code availability

• The datasets generated during this study are publicly available at Figshare: https://doi.org/10.6084/m9.figshare.32049963.
• The code supporting this study is available from the corresponding author upon reasonable request.
• Any additional information required to reanalyze the data reported in this study is available from the lead contact upon request.

## Acknowledgments

This work was funded by the 10.13039/501100005645Ministry of Education and Training, under grant no. B2026-SPK-03, and hosted by Ho Chi Minh City University of Technology and Engineering, Vietnam.

## Author contributions

T.N.N., data curation, formal analysis, methodology, resources, software, visualization, writing – original draft, and writing – review and editing; C.C.V., conceptualization, formal analysis, funding acquisition, investigation, methodology, project administration, resources, software, supervision, validation, visualization, writing – original draft, and writing – review and editing; V.H.N., formal analysis, software, and visualization; V.-C.P., conceptualization, formal analysis, resources, validation, and writing – review and editing.

## Declaration of interests

The authors declare no competing interests.

## STAR★Methods

### Key resources table

**Table:** 

REAGENT or RESOURCE	SOURCE	IDENTIFIER
**Chemicals, peptides, and recombinant proteins**		

Carbon nanotubes/textiles	CloApha, Seoul, South Korea	Commercial supplier

**Deposited data**		
Object classification dataset	Figshare	Figshare: https://doi.org/10.6084/m9.figshare.32049963

**Software and algorithms**		
TensorFlow Lite for Microcontrollers	Google	https://www.tensorflow.org/lite/microcontrollers
Scikit-learn	Python package	https://scikit-learn.org
Arduino IDE	Arduino	https://www.arduino.cc
Python	Python Software Foundation	https://www.python.org
MATLAB	MathWorks	https://www.mathworks.com

**Other**		
Au electrodes	Thien Lam Electronic, Ho Chi Minh City, Viet Nam	Commercial supplier
3M adhesive tape	3M, Minnesota, United States	Commercial supplier
ESP32S3 microcontroller	Seeed Studio, Shenzhen, China	XIAO ESP32S3

### Experimental model and study participant details

Three healthy male volunteers aged 23–35 years participated in the object-grasping experiments. All participants were right-handed and reported no hand-related musculoskeletal or neurological disorders. Data were collected under controlled indoor conditions. Each participant performed grasping actions for six object categories, and samples were distributed approximately evenly among participants and object classes.

All participants provided informed consent prior to data collection. The study was conducted in accordance with institutional guidelines.

Only male participants were included in this proof-of-concept study. Therefore, potential sex- or gender-related differences in grasping behavior and pressure distribution were not evaluated and represent a limitation of the current work. Future studies will include a larger and more diverse participant population to assess generalizability across sex and demographic groups.

### Method details

#### Fabrication of textile pressure sensors

The textile pressure sensors were fabricated by depositing CNT-based conductive materials onto thin flexible fabric substrates. Au electrodes with an active sensing area of approximately 10 × 8 mm^2^ were patterned on the substrate surface and connected to conductive sensing layers to form pressure-sensitive structures. The fabricated sensors were attached to the thumb, index, and middle fingers of a wearable glove platform for object-grasping measurements.

#### Sensor characterization

The morphology of the sensing materials and electrode structures was characterized using scanning electron microscopy. Electrical responses were evaluated under repeated loading and unloading conditions. The fabricated sensor exhibited a sensitivity of 0.46 kPa^−1^, a response time of 60 ms, and a recovery time of 45 ms. Durability tests were conducted for approximately 3500 loading cycles under identical experimental conditions. Five independently fabricated sensors were tested, and averaged values were reported with standard deviation error bars.

#### Data acquisition and preprocessing

Pressure signals from the thumb, index, and middle finger sensors were collected using an ESP32S3 microcontroller equipped with a 12-bit analog-to-digital converter. The sampling frequency was set to 100 Hz. Each sensing window contained 250 time points per channel over a duration of 2.5 s, resulting in an input tensor size of 250 × 3 for classification. The collected signals were normalized prior to feature extraction and machine learning analysis.

#### TinyML model training and deployment

Random forest and k-nearest neighbors models were implemented using Python and Scikit-learn libraries for object classification. The dataset was divided into training and testing subsets using an 60:40 ratio. The random forest and k-nearest neighbors models achieved classification accuracies of 92.1% and 96.55%, respectively. The trained lightweight models were deployed on the ESP32S3 embedded platform for real-time on-device inference.

#### Real-time embedded inference

The smart glove system performed local inference directly on the ESP32S3 platform. The random forest and k-nearest neighbors models achieved embedded inference times of approximately 0.185 ms and 4 ms, respectively. Only classification labels and inference results were transmitted through Wi-Fi communication, while raw sensing data remained on the embedded device. Real-time performance was evaluated using embedded inference latency, memory usage, and classification accuracy.

### Quantification and statistical analysis

Five independently fabricated sensors were tested under identical conditions. Reported values represent mean performance, and error bars indicate standard deviation.

### Additional resources

No additional resources are associated with this study.

## Supplemental information

Document S1. Figures S1–S4 and Tables S1–S2

Video S1. Real-time demonstration of object classification using the smart glove with on-device TinyML inference
