# Supplementary material for: Thin fabric pressure sensors and TinyML smart gloves for edge IoT
Source: iScience. 2026 Aug 1;29(8):116750. doi: 10.1016/j.isci.2026.116750 (PMC13453505; doi:10.1016/j.isci.2026.116750)
Supplement: Supplementary file 1 [file mmc1.pdf]

**iScience, Volume *29***

## Supplemental information

# Thin fabric pressure sensors and TinyML smart gloves for edge IoT

**Tuan Nghia Nguyen, Chi Cuong Vu, Viet Hoang Nguyen, and Van-Ca Phan**

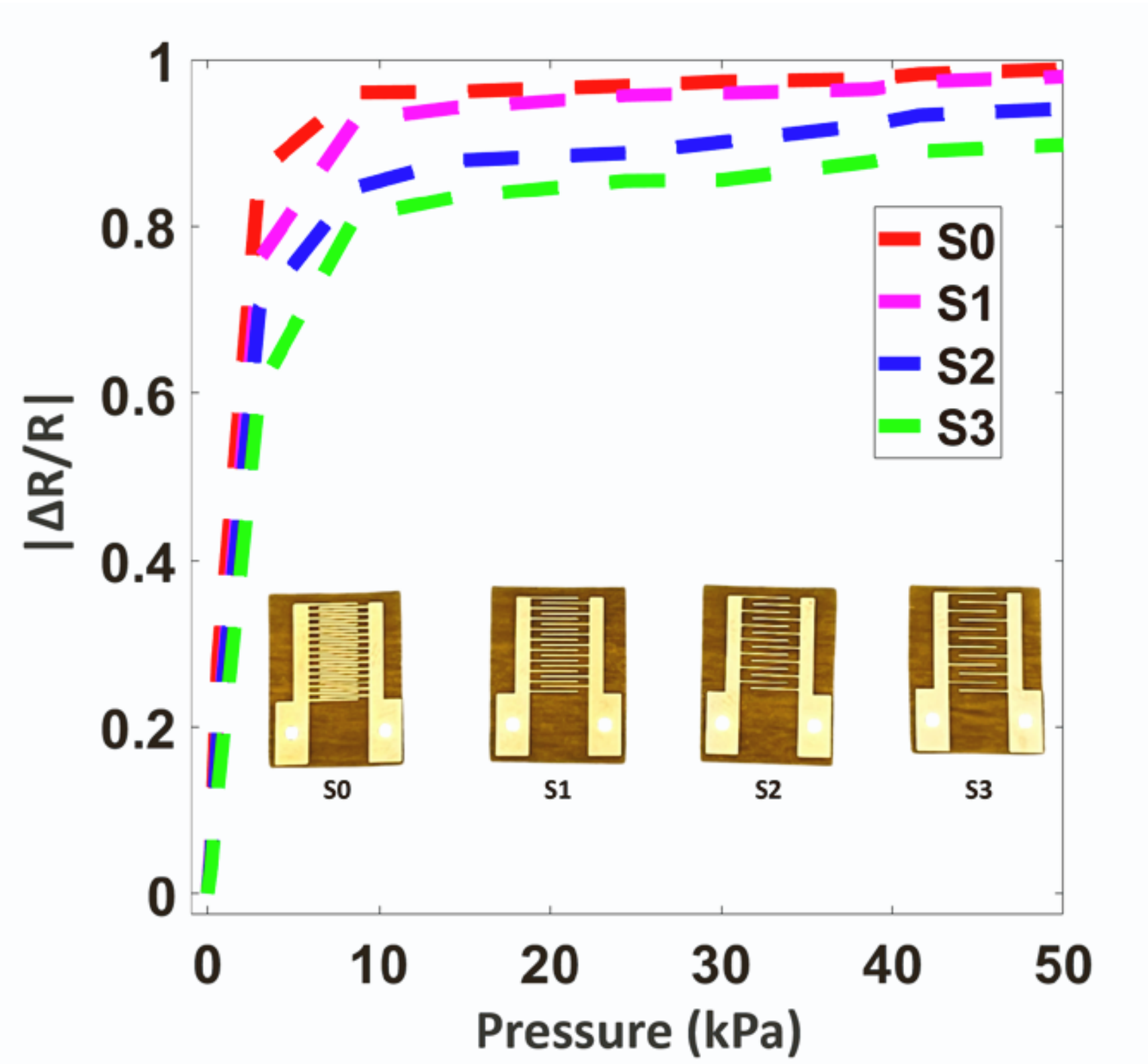


**Figure S1.** The change in electrical resistance of different electrode structures.

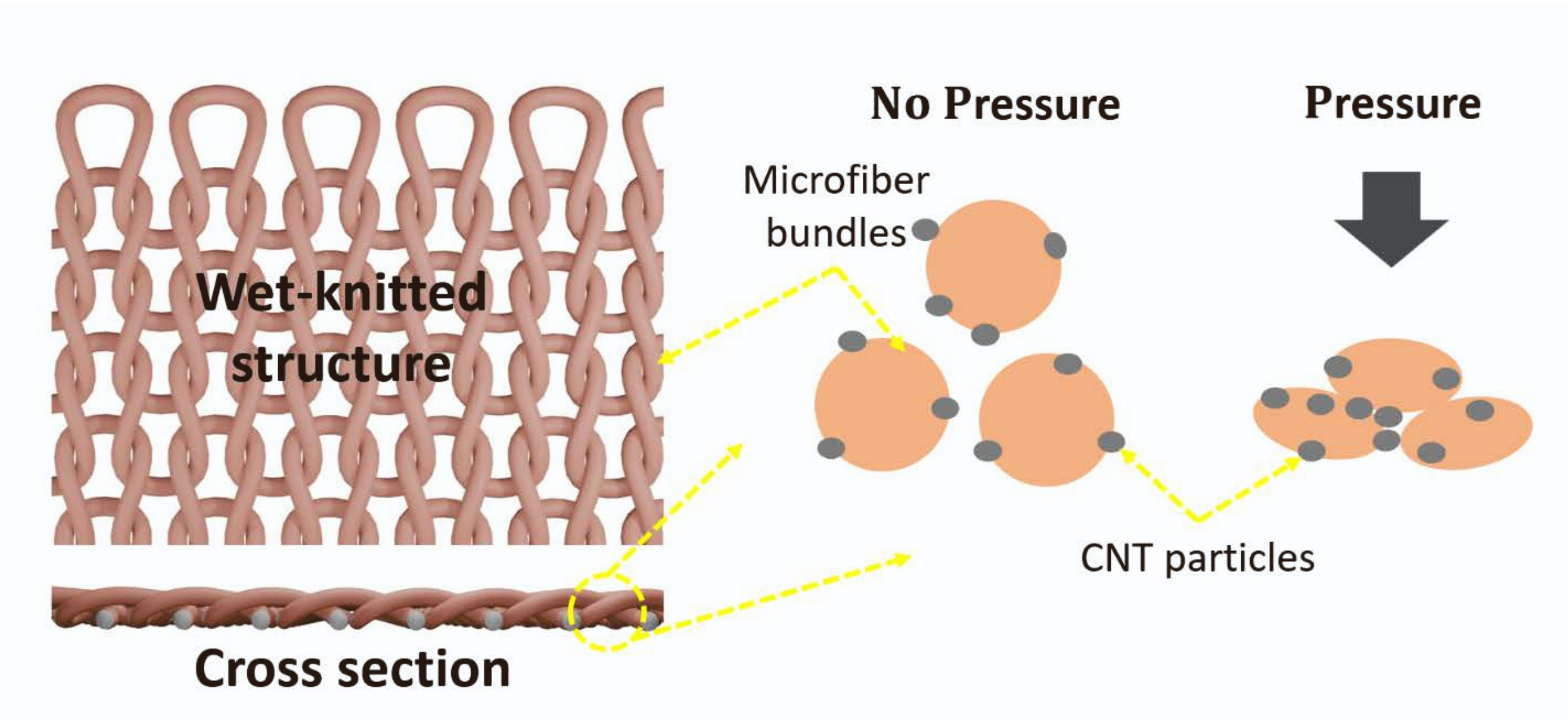


**Figure S2.** The operating principle of the CNT-knitted fabric under pressure.

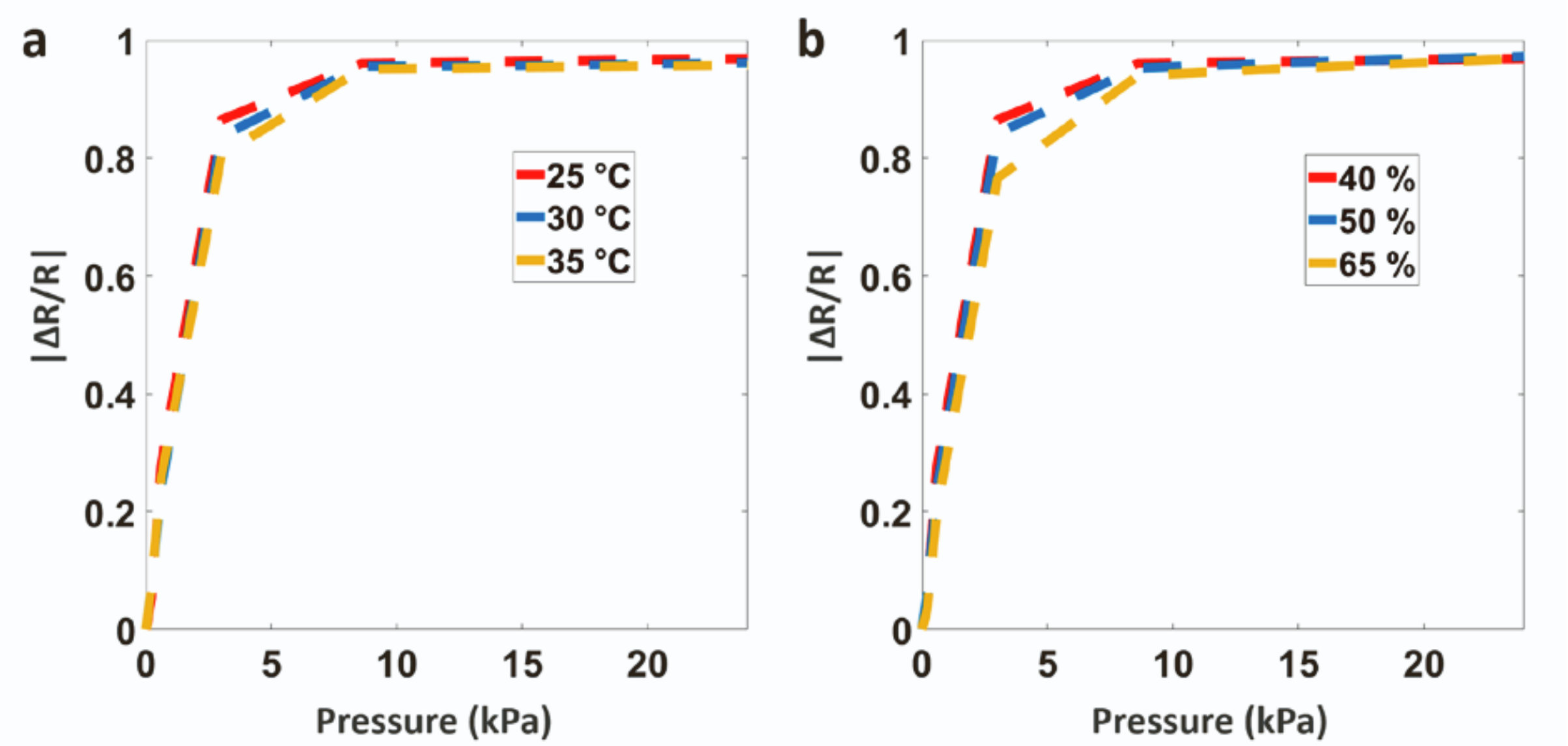


**Figure S3.** Pressure-dependent response of the sensor under (a) Different temperatures (25–30–35 °C) and (b) Different humidity levels (40–50–65%).

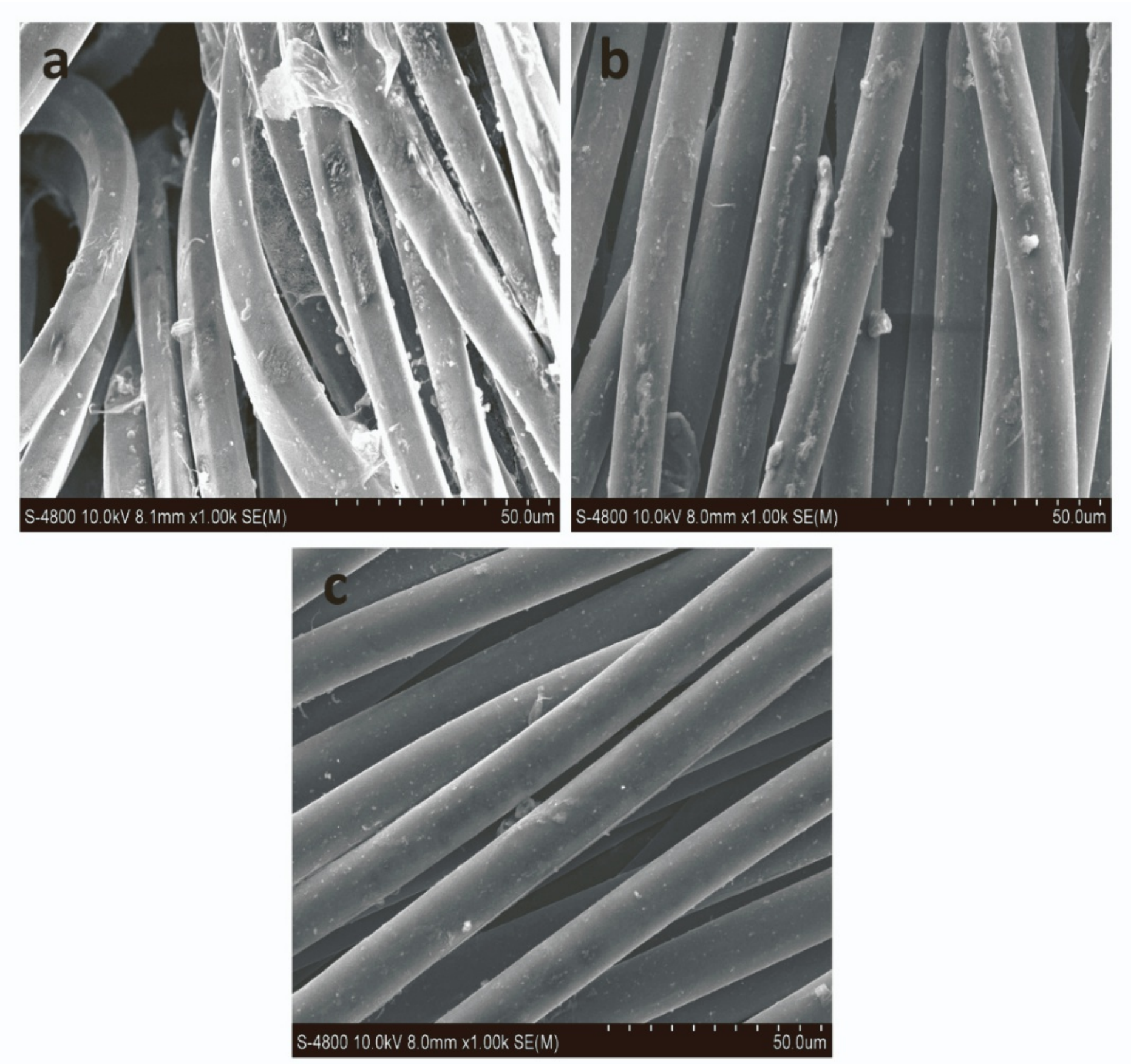


**Figure S4.** SEM images of the CNT-fabric layer after cyclic durability tests: (a) 0 cycles, (b) 5,000 cycles, and (c) 10,000 cycles.

**Table S1.** Wearability and motion-interference evaluation.

| Task | Condition | Completion time (s) | Success rate (%) | Failed grasps (count) | ROM proxy (%) |
|---|---|---|---|---|---|
| Bottle grasp | Bare hand | 1.95 ± 0.22 | 100 | 0 | 100 |
| | Glove | 2.20 ± 0.25 | 100 | 0 | 97 |
| Cup pick-up | Bare hand | 2.20 ± 0.30 | 100 | 0 | 100 |
| | Glove | 2.35 ± 0.33 | 95 | 1 | 96 |
| Soft object | Bare hand | 2.25 ± 0.35 | 100 | 0 | 100 |
| | Glove | 2.38 ± 0.40 | 95 | 1 | 95 |

**Table S2.** Power consumption under different operating modes.

| Mode | Current (mA) | Power (mW) |
|---|---|---|
| Idle (Wi-Fi connected) | 59 | 218.3 |
| Sensor acquisition active | 63 | 233.1 |
| Preprocessing active | 66 | 244.2 |
| Inference active | 67-70 | 247.9-259 |
| Full operation | 70.3 | 260.1 |
